# Crystal structure of 2-chloro-5-(3-hy­droxy-3-methyl­but-1-yn-1-yl)pyrimidine

**DOI:** 10.1107/S2056989017010027

**Published:** 2017-07-13

**Authors:** Jörg Hübscher, Wilhelm Seichter, Edwin Weber

**Affiliations:** a Institut für Organische Chemie, TU Bergakademie Freiberg, Leipziger Strasse 29, D-09596 Freiberg/Sachsen, Germany

**Keywords:** crystal structure, 5-ethynyl­pyrimidine derivative, O—H⋯N hydrogen bonding, C—H⋯O contact

## Abstract

In the title compound, C_9_H_9_ClN_2_O, the ethynyl­pyrimidine moiety displays an almost planar geometry. In the crystal, mol­ecules are linked by O—H⋯N and C—H_pyrimidine_⋯O hydrogen bonds, forming a three-dimensional supra­molecular architecture.

## Abstract 2

The ethynyl­pyrimidine moiety displays an almost planar geometry. In the crystal, mol­ecules are linked by O—H⋯N and C—H_pyrimidine_⋯O hydrogen bonds, forming a three-dimensional supra­molecular architecture.

## Chemical context   

The title compound, featuring a blocked acetyl­enic group and a chloro-substituted pyrimidine ring, is an inter­esting synthetic inter­mediate for the preparation of application-oriented solid materials including both porous coordination polymers (MacGillivray, 2010 ▸) and metal-organic frameworks (Noro & Kitagawa, 2010 ▸). Deprotection of the acetyl­enic functional group and transformation of the chloro substituent, *e.g*. into thiol or amino groups, should result in mol­ecular building blocks for the formation of corresponding aggregate structures (Hübscher *et al.*, 2015 ▸; Günthel *et al.*, 2015 ▸; Hübscher *et al.*, 2017 ▸). Aside from this experimental preparative relevance, substituted 3-hy­droxy­alkynes are also of considerable inter­est due to their structural capacity in supra­molecular inter­actions, giving rise to particular modes of aggregation and behavior in the solid state (Toda *et al.*, 1983 ▸, 1985 ▸; Bourne *et al.*, 1994 ▸). In combination with heterocyclic nitro­gen donors and chlorine substitution, as in the present title compound, a structural study involving competition aspects with regard to hydrogen bonding (Wang & Zheng, 2015 ▸) and potential halogen (Mukherjee *et al.*, 2014 ▸) or π-electron assisted (Tiekink & Zukerman-Schpector, 2012 ▸) inter­actions should be a promising field of inquiry for crystal engineering (Desiraju *et al.*, 2012 ▸) being subject to the contacts emanating from a variety of functional groups. Thus, in this respect, the title compound could serve as a worthwhile test substance.
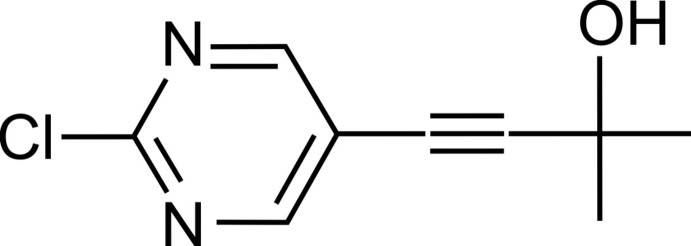


## Structural commentary   

**Figure 1:**
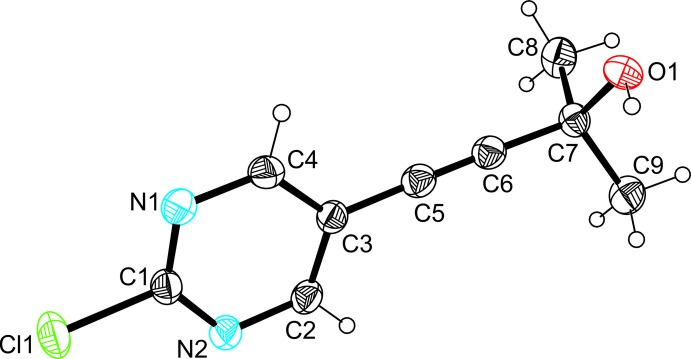
Perspective view of the mol­ecular structure of the title compound including the atom-numbering scheme. Displacement parameters are drawn at the 50% probability level.

A perspective view of the mol­ecular structure of the title compound is depicted in Fig. 1 ▸. The ethynyl­pyrimidine moiety of the mol­ecule is almost planar with the largest atomic distances from the mean plane being 0.015 (1) Å for atom C1 and 0.013 (1) Å for atom C4. The OH group adopts a staggered arrangement with respect to the ethynyl unit and the methyl group C9, the C6—C7—O1–H1 torsion angle being 57.0°.

## Supra­molecular features   

**Figure 2:**
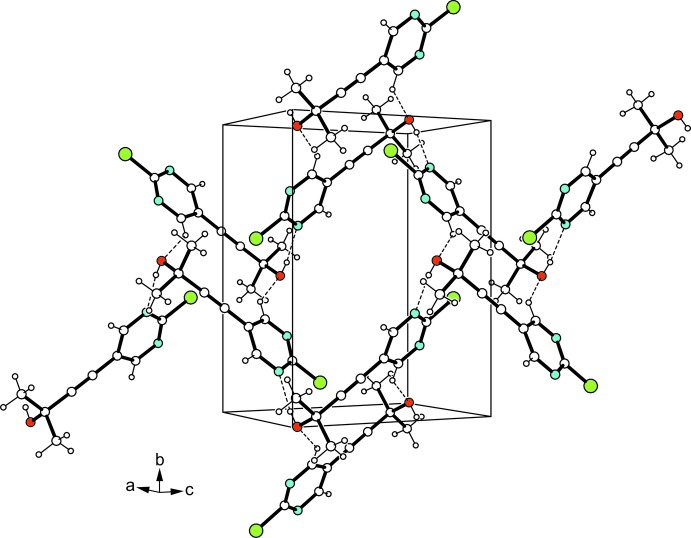
Packing excerpt of the title compound. Hydrogen bonds are shown as dashed lines.

An O—H⋯π_C≡C_ hydrogen-bond type inter­molecular inter­action mode typical of 3-hy­droxy­alkyne structure units (Desiraju & Steiner, 1999 ▸) is not present here, apparently in favor of a stronger O—H⋯N hydrogen bond involving the hy­droxy group and a pyrimidine nitro­gen atom (N2). Aside from this, C—H_pyrimidine_⋯O hydrogen bonds are found to yield a three-dimensional supra­molecular architecture (Table 1 ▸, Fig. 2 ▸). No other types of directed inter­molecular contacts, including those involving the Cl atom or π–arene stacking, are observed. Hence, this shows that in the presence of a strong donor center such as a nitro­gen atom, competing with the acetyl­enic moiety, the common O—H⋯π_C≡C_ hydrogen bonding is suppressed, which could be a useful finding in relation to aspects of crystal engineering.

## Database survey   

The title compound represents the first example of a 5-(3-hy­droxy-3-methyl­but-1-yn-1-yl)pyrimidine. A search in the Cambridge Structural Database (CSD, Version 5.38, update February 2017; Groom *et al.*, 2016 ▸) for compounds containing the 4-ethynyl­pyrimidine fragment excluding metal complexes and co-crystals revealed nine hits. Of particular inter­est is the crystal structure of 5,5′-ethyne-1,2-diylbis(2-chloro­pyrimidine) (refcode: PUMHIQ; Hübscher *et al.*, 2015 ▸). In this case, the absence of a strongly coordinating donor/acceptor substituent results in poor mol­ecular association, which is restricted to π_pyrimidine_⋯π_ethyne_ stacking inter­actions.

## Synthesis and crystallization   

The title compound was prepared from 2-hy­droxy-5-iodo­pyrimidine (Pérez-Palado *et al.*, 2007 ▸) and 2-methyl-3-butyn-2-ol (MEBYNOL) *via* a Shonogashira–Hagihara cross-coupling reaction (Sonogashira *et al.*, 1975 ▸) as follows. 2-Chloro-5-iodo­pyrimidine (2.0 g, 8.4 mmol) and MEBYNOL (0.7 g, 8.7 mmol) were dissolved in a degassed mixture of dry diiso­propyl­amine and THF (60 ml each). To this solution, the catalyst being composed of tri­phenyl­phosphine (2 mol%), copper(I) and iodide (3 mol-%) and *trans*-di­chloro­bis­(tri­phenyl­phosphine)palladium(II) (2 mol%) was added. The mixture was stirred at room temperature away from light for 12 h, then filtered over Celite and evaporated. Crystallization from *n*-hexane gave colourless crystals of the title compound on slow evaporation of the solvent (yield 1.1 g, 70%; m.p. 455 K). ^1^H NMR (CDCl_3_): δ_H_ 8.64 (2H, *s*, pyr-H), 2.67 (1H, *s*, OH), 1.64 (6H, *s*, Me). ^13^C NMR (CDCl_3_): δ_C_ 161.1 (pyrC-4), 159.4 (pyrC-2), 117.8 (pyrC-5), 102.4 (pyr-*C*≡C), 74.1 (pyr-C≡*C*), 65.5 (C_quat._), 31.1 (CH_3_). IR (KBr) ν_max._ 2240 (C≡C). GC–MS: calculated for C_9_H_9_N_2_OCl (196.04), found 196 [*M*]^+^. Analysis calculated for C_9_H_9_N_2_OCl: C, 54.97; H, 4.61; N, 14.25; found: C, 54.81; H, 4.56; N, 14.05%. Colourless crystals suitable for X-ray diffraction were obtained by slow evaporation of solvent from a chloro­form solution.

## Refinement   

Crystal data, data collection and structure refinement details are summarized in Table 2 ▸. H atoms were included in calculated positions (C—H = 0.95, 0.98 Å; O—H = 0.84 Å) and allowed to ride on their parent atoms with *U*_iso_(H) = 1.5*U*_eq_(C,O) for methyl and hy­droxy H atoms and 1.2*U*_eq_(C) for aryl H atoms.

## Supplementary Material

Crystal structure: contains datablock(s) I. DOI: 10.1107/S2056989017010027/zq2237sup1.cif

Structure factors: contains datablock(s) I. DOI: 10.1107/S2056989017010027/zq2237Isup2.hkl

Supporting information file. DOI: 10.1107/S2056989017010027/zq2237Isup3.cml

CCDC reference: 1560599

Additional supporting information:  crystallographic information; 3D view; checkCIF report

## Figures and Tables

**Table 1:** Hydrogen-bond geometry (Å, °)

*D*—H⋯*A*	*D*—H	H⋯*A*	*D*⋯*A*	*D*—H⋯*A*
C8—H8*A*⋯Cl1^i^	0.98	2.99	3.7911 (14)	140
C4—H4⋯O1^ii^	0.95	2.45	3.1963 (15)	136
C2—H2⋯O1^iii^	0.95	2.60	3.2816 (14)	129
O1—H1⋯N2^iv^	0.84	2.05	2.8881 (13)	172

Symmetry codes: (i) 
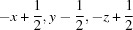
; (ii) 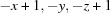; (iii) 
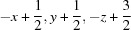
; (iv) 
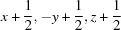
.

**Table 2:** Experimental details

Crystal data	
Chemical formula	C_9_H_9_ClN_2_O
*M*_r_	196.63
Crystal system, space group	Monoclinic, *P*2_1_/*n*
Temperature (K)	153
*a*, *b*, *c* (Å)	7.5555 (3), 13.0278 (7), 9.7397 (5)
β (°)	91.767 (2)
*V* (Å^3^)	958.24 (8)
*Z*	4
Radiation type	Mo *K*α
μ (mm^−1^)	0.36
Crystal size (mm)	0.60 × 0.60 × 0.20

Data collection	
Diffractometer	Bruker X8 *APEX2* CCD detector
Absorption correction	Multi-scan (*SADABS*; Bruker, 2008 ▸)
*T*_min_, *T*_max_	0.814, 0.932
No. of measured, independent and observed [*I* > 2σ(*I*)] reflections	8464, 1988, 1795
*R*_int_	0.021
(sin θ/λ)_max_ (Å^−1^)	0.628

Refinement	
*R*[*F* ^2^ > 2σ(*F* ^2^)], *wR*(*F* ^2^), *S*	0.027, 0.074, 1.07
No. of reflections	1988
No. of parameters	121
H-atom treatment	H-atom parameters constrained
Δρ_max_, Δρ_min_ (e Å^−3^)	0.22, −0.26

Computer programs: *APEX2* and *SAINT* (Bruker, 2008 ▸), *SHELXS97* and *SHELXTL* (Sheldrick, 2008 ▸), *SHELXL2015* (Sheldrick, 2015 ▸) and *ORTEP-3 for Windows* (Farrugia, 2012 ▸).

## supplementary crystallographic information

### Crystal data

**Table:**

C_9_H_9_ClN_2_O	*F*(000) = 408
*M**_r_* = 196.63	*D*_x_ = 1.363 Mg m^−^^3^
Monoclinic, *P*2_1_/*n*	Mo *K*α radiation, λ = 0.71073 Å
*a* = 7.5555 (3) Å	Cell parameters from 5586 reflections
*b* = 13.0278 (7) Å	θ = 2.6–29.2°
*c* = 9.7397 (5) Å	µ = 0.36 mm^−^^1^
β = 91.767 (2)°	*T* = 153 K
*V* = 958.24 (8) Å^3^	Block, colourless
*Z* = 4	0.60 × 0.60 × 0.20 mm

### Data collection

**Table:**

Bruker X8 APEX2 CCD detector diffractometer	1795 reflections with *I* > 2σ(*I*)
φ and ω scans	*R*_int_ = 0.021
Absorption correction: multi-scan (SADABS; Bruker, 2008)	θ_max_ = 26.5°, θ_min_ = 2.6°
*T*_min_ = 0.814, *T*_max_ = 0.932	*h* = −9→9
8464 measured reflections	*k* = −15→16
1988 independent reflections	*l* = −10→12

### Refinement

**Table:**

Refinement on *F*^2^	0 restraints
Least-squares matrix: full	Hydrogen site location: inferred from neighbouring sites
*R*[*F*^2^ > 2σ(*F*^2^)] = 0.027	H-atom parameters constrained
*wR*(*F*^2^) = 0.074	*w* = 1/[σ^2^(*F*_o_^2^) + (0.0363*P*)^2^ + 0.3033*P*] where *P* = (*F*_o_^2^ + 2*F*_c_^2^)/3
*S* = 1.07	(Δ/σ)_max_ < 0.001
1988 reflections	Δρ_max_ = 0.22 e Å^−^^3^
121 parameters	Δρ_min_ = −0.26 e Å^−^^3^

### Special details

**Table:**

Experimental. The melting point was measured using a microscope heating stage (Thermovar, Reichert–Jung). The NMR spectra were obtained on a Bruker Avance 500.1 (^1^H) and 125.8 MHz (^13^C) with TMS as internal standard (δ in ppm). The IR spectrum was determined on a Nicolet FT–IR 510 spectrometer as KBr pellet (wavenumber is given in cm^-1^). The mass spectrum was recorded on a Hewlett–Packard 5890 Series II/MS 5989A. Elemental analysis was carried out with a Hanau vario MICRO cube.
Geometry. All esds (except the esd in the dihedral angle between two l.s. planes) are estimated using the full covariance matrix. The cell esds are taken into account individually in the estimation of esds in distances, angles and torsion angles; correlations between esds in cell parameters are only used when they are defined by crystal symmetry. An approximate (isotropic) treatment of cell esds is used for estimating esds involving l.s. planes.

### Fractional atomic coordinates and isotropic or equivalent isotropic displacement parameters (Å^2^)

**Table:**

	*x*	*y*	*z*	*U*_iso_*/*U*_eq_	
Cl1	0.23192 (4)	0.39748 (3)	0.09166 (3)	0.03135 (12)	
O1	0.40922 (11)	−0.02601 (6)	0.82873 (9)	0.0229 (2)	
H1	0.4836	0.0217	0.8368	0.034*	
N1	0.33701 (13)	0.23553 (8)	0.22655 (10)	0.0237 (2)	
N2	0.14057 (13)	0.34952 (8)	0.33846 (10)	0.0227 (2)	
C1	0.23671 (15)	0.31831 (9)	0.23470 (12)	0.0205 (2)	
C2	0.14420 (15)	0.28822 (9)	0.44881 (12)	0.0219 (3)	
H2	0.0768	0.3066	0.5258	0.026*	
C3	0.24367 (15)	0.19835 (9)	0.45432 (12)	0.0196 (2)	
C4	0.34057 (16)	0.17568 (9)	0.33843 (12)	0.0228 (3)	
H4	0.4117	0.1155	0.3389	0.027*	
C5	0.24486 (15)	0.13360 (10)	0.57316 (12)	0.0222 (3)	
C6	0.24267 (15)	0.08049 (9)	0.67312 (12)	0.0221 (3)	
C7	0.23766 (15)	0.01625 (9)	0.79857 (12)	0.0205 (2)	
C8	0.11447 (17)	−0.07507 (11)	0.77342 (14)	0.0292 (3)	
H8A	0.1610	−0.1182	0.7004	0.044*	
H8B	−0.0037	−0.0503	0.7457	0.044*	
H8C	0.1070	−0.1154	0.8580	0.044*	
C9	0.17864 (17)	0.08197 (11)	0.91878 (13)	0.0281 (3)	
H9A	0.1825	0.0408	1.0030	0.042*	
H9B	0.0574	0.1062	0.9003	0.042*	
H9C	0.2581	0.1410	0.9300	0.042*	

### Atomic displacement parameters (Å^2^)

**Table:**

	*U*^11^	*U*^22^	*U*^33^	*U*^12^	*U*^13^	*U*^23^
Cl1	0.03714 (19)	0.0336 (2)	0.02359 (18)	0.00504 (13)	0.00606 (13)	0.01072 (12)
O1	0.0209 (4)	0.0193 (4)	0.0288 (5)	0.0002 (3)	0.0028 (3)	0.0023 (3)
N1	0.0271 (5)	0.0234 (5)	0.0209 (5)	0.0017 (4)	0.0062 (4)	−0.0002 (4)
N2	0.0255 (5)	0.0229 (5)	0.0197 (5)	0.0039 (4)	0.0020 (4)	0.0003 (4)
C1	0.0221 (5)	0.0213 (6)	0.0180 (6)	−0.0013 (4)	0.0010 (4)	0.0017 (4)
C2	0.0238 (6)	0.0246 (6)	0.0174 (6)	0.0019 (5)	0.0031 (4)	−0.0024 (5)
C3	0.0211 (5)	0.0200 (6)	0.0176 (6)	−0.0021 (4)	0.0003 (4)	−0.0005 (4)
C4	0.0265 (6)	0.0194 (6)	0.0228 (6)	0.0024 (5)	0.0043 (5)	−0.0004 (5)
C5	0.0228 (6)	0.0224 (6)	0.0215 (6)	0.0003 (5)	0.0033 (5)	−0.0015 (5)
C6	0.0231 (6)	0.0222 (6)	0.0213 (6)	0.0007 (5)	0.0041 (5)	−0.0007 (5)
C7	0.0202 (5)	0.0224 (6)	0.0189 (6)	−0.0001 (4)	0.0032 (4)	0.0023 (5)
C8	0.0298 (6)	0.0314 (7)	0.0264 (6)	−0.0090 (5)	0.0016 (5)	0.0032 (5)
C9	0.0287 (6)	0.0330 (7)	0.0230 (6)	0.0040 (5)	0.0075 (5)	0.0000 (5)

### Geometric parameters (Å, º)

**Table:**

Cl1—C1	1.7329 (12)	C4—H4	0.9500
O1—C7	1.4304 (14)	C5—C6	1.1950 (18)
O1—H1	0.8400	C6—C7	1.4824 (16)
N1—C1	1.3219 (16)	C7—C8	1.5257 (17)
N1—C4	1.3396 (16)	C7—C9	1.5280 (17)
N2—C1	1.3265 (16)	C8—H8A	0.9800
N2—C2	1.3386 (15)	C8—H8B	0.9800
C2—C3	1.3914 (17)	C8—H8C	0.9800
C2—H2	0.9500	C9—H9A	0.9800
C3—C4	1.3958 (17)	C9—H9B	0.9800
C3—C5	1.4320 (16)	C9—H9C	0.9800
			
C7—O1—H1	109.5	O1—C7—C8	106.10 (10)
C1—N1—C4	115.04 (10)	C6—C7—C8	109.81 (10)
C1—N2—C2	115.51 (10)	O1—C7—C9	110.02 (9)
N1—C1—N2	128.63 (11)	C6—C7—C9	109.31 (10)
N1—C1—Cl1	115.79 (9)	C8—C7—C9	111.65 (10)
N2—C1—Cl1	115.58 (9)	C7—C8—H8A	109.5
N2—C2—C3	122.05 (11)	C7—C8—H8B	109.5
N2—C2—H2	119.0	H8A—C8—H8B	109.5
C3—C2—H2	119.0	C7—C8—H8C	109.5
C2—C3—C4	116.30 (11)	H8A—C8—H8C	109.5
C2—C3—C5	121.11 (11)	H8B—C8—H8C	109.5
C4—C3—C5	122.59 (11)	C7—C9—H9A	109.5
N1—C4—C3	122.47 (11)	C7—C9—H9B	109.5
N1—C4—H4	118.8	H9A—C9—H9B	109.5
C3—C4—H4	118.8	C7—C9—H9C	109.5
C6—C5—C3	178.65 (13)	H9A—C9—H9C	109.5
C5—C6—C7	178.79 (13)	H9B—C9—H9C	109.5
O1—C7—C6	109.91 (9)		
			
C4—N1—C1—N2	0.20 (19)	N2—C2—C3—C4	0.30 (17)
C4—N1—C1—Cl1	179.40 (9)	N2—C2—C3—C5	−179.66 (11)
C2—N2—C1—N1	−0.89 (19)	C1—N1—C4—C3	0.82 (17)
C2—N2—C1—Cl1	179.91 (8)	C2—C3—C4—N1	−1.05 (18)
C1—N2—C2—C3	0.58 (17)	C5—C3—C4—N1	178.91 (11)

### Hydrogen-bond geometry (Å, º)

**Table:**

*D*—H···*A*	*D*—H	H···*A*	*D*···*A*	*D*—H···*A*
C8—H8*A*···Cl1^i^	0.98	2.99	3.7911 (14)	140
C4—H4···O1^ii^	0.95	2.45	3.1963 (15)	136
C2—H2···O1^iii^	0.95	2.60	3.2816 (14)	129
O1—H1···N2^iv^	0.84	2.05	2.8881 (13)	172

Symmetry codes: (i) −*x*+1/2, *y*−1/2, −*z*+1/2; (ii) −*x*+1, −*y*, −*z*+1; (iii) −*x*+1/2, *y*+1/2, −*z*+3/2; (iv) *x*+1/2, −*y*+1/2, *z*+1/2.
